# Impaired Fracture Healing Caused by Deficiency of the Immunoreceptor Adaptor Protein DAP12

**DOI:** 10.1371/journal.pone.0128210

**Published:** 2015-06-01

**Authors:** Masayuki Kamimura, Yu Mori, Akiko Sugahara-Tobinai, Toshiyuki Takai, Eiji Itoi

**Affiliations:** 1 Department of Orthopaedic Surgery, Tohoku University Graduate School of Medicine 1–1 Seiryo-machi, Aobaku, Sendai, Miyagi, Japan; 2 Department of Experimental Immunology, Institute of Development, Aging and Cancer, Tohoku University4-1 Seiryo-machi, Aobaku, Sendai, Miyagi, Japan; Université de Lyon - Université Jean Monnet, FRANCE

## Abstract

Osteoclasts play an important role in bone metabolism, but their exact role in fracture healing remains unclear. DAP12 is an immunoadaptor protein with associated immunoreceptors on myeloid lineage cells, including osteoclasts. Its deficiency causes osteopetrosis due to suppression of osteoclast development and activation. In this report, we assessed the impact of DAP12 on the fracture healing process using C57BL/6 (B6) and *DAP12^–/–^* mice. Healing was evaluated using radiography, micro-CT, histology, immunohistochemistry and real-time RT-PCR. Radiography showed lower callus volume and lower callus radiolucency in *DAP12^–/–^* mice during later stages. Micro-CT images and quantitative structural analysis indicated that *DAP12^–/–^* mice developed calluses of dense trabecular structures and experienced deteriorated cortical shell formation on the surface. Histologically, *DAP12^–/–^* mice showed less cartilaginous resorption and woven bone formation. In addition, prominent cortical shell formation was much less in *DAP12^–/–^* mice. Immunohistochemistry revealed lower invasion of F4/80 positive monocytes and macrophages into the fracture hematoma in *DAP12^–/–^* mice. The expression levels of *Col1a1*, *Col2a1* and *Col10a1* in *DAP12^–/–^* mice increased and subsequently became higher than those in B6 mice. There was a decrease in the gene expression of *Tnf* during the early stages in *DAP12^–/–^* mice. Our results indicate that DAP12 deficiency impairs fracture healing, suggesting a significant role of DAP12 in the initial inflammatory response, bone remodeling and regeneration.

## Introduction

Post-menopausal osteoporosis is a pathological condition that is caused by a deficiency in estrogen and that induces rapid bone resorption; the subsequent osteoporosis increases fracture risk. There are many effective anti-bone resorption drugs, but osteoporotic fractures remain a serious global health problem [1,2]. Bisphosphonates, inhibitors of bone resorption through osteoclast suppression, are the most widely used agents to treat osteoporosis and prevent osteoporotic fractures. However, bisphosphonates inhibit bone turnover including bone resorption and subsequent bone formation [3]. In addition, long-term use of bisphosphonates is associated with an increased risk of atypical hip fractures [4]. Several studies using animal fracture models showed that bisphosphonate treatment impaired callus remodeling and maturation during fracture repair [5–7]. Osteoclasts play an important role in bone metabolism; however, their detailed mechanism in fracture healing remains unclear.

DNAX-activation protein 12 (DAP12) is a 12 kDa adaptor protein that contains an immunoreceptor tyrosine-based activation motif (ITAM) and is expressed on myeloid lineage cells including osteoclasts [8]. DAP12 associates with various immunoreceptors and with triggering receptor expressed on myeloid cells-2 (TREM2). Phosphorylation of ITAM stimulated by RANKL-RANK binding and immunoreceptor ligation results in the recruitment of spleen tyrosine kinases (SYKs), leading to the activation of phospholipase C gamma (PLC-γ) and calcium signaling, which is critical for auto-amplification of the nuclear factor of activated T-cells, cytoplasmic 1 (NFATc1) and induction of osteoclast-specific genes [9]. Deletions or inactivating mutations in human DAP12 or DAP12-associated immunoreceptor TREM2 is associated with Nasu-Hakola disease, characterized by multiple bony cysts in cancellous bone leading to pathological fractures and arthritis, as well as fatal neurodegenerative disorders accompanying severe dementia [10–12]. Kaifu et al. reported that DAP12-deficient mice showed inhibited osteoclast development *in vitro* and osteopetrosis and thalamic hypomyelinosis *in vivo* [13]. Patients with Nasu-Hakola disease are vulnerable to fracture; however, it is unclear whether osteoclast suppression in these patients induces impaired fracture healing. To characterize the role of osteoclasts in fracture healing, many studies have investigated fracture repair using various osteoclast-inhibited animal models and strong anti-bone resorption reagents [5–7,14–19]. The effects of anti-bone resorption reagents on fracture healing have been investigated by administration of pamidronate, incadronate, zoledronate, and osteoprotegerin. However, in some studies, the dose of drugs administered was above the physiological limits. Fracture studies on naturally-occurring osteopetrotic animals have utilized rats lacking incisors. Most studies have indicated increasing callus volume and delayed bone remodeling, and concluded that osteoclast function may not be critical for the early phases of soft callus union, but is essential for hard callus remodeling. DAP12 is expressed not only in osteoclasts, but also in macrophages and other cells of myeloid lineage [8]. Therefore, DAP12 may be associated with inflammation in the early phase of fracture healing process. Although DAP12 plays an important role in the differentiation and function of osteoclasts, the effect of DAP12-deficiency on fracture healing is not well understood.

In the current study, we hypothesized that DAP12 impairment affects both soft callus formation and hard callus remodeling, and that different radiological, histological and molecular biological patterns would be observed between C57BL/6 (B6) mice and DAP12-deficient (*DAP12* ^*–/–*^) mice during the healing process. To test this hypothesis, we assessed bone healing in murine tibia fractures in B6 and *DAP12* ^*–/–*^ mice using radiological, histological and gene expression analyses.

## Material and Methods

### Animals

*DAP12* ^*–/–*^ mice were generated using 129/SvJ and C57BL/6 (B6) mice as described previously [20] and backcrossed onto B6 mice for 15 generations. Wild-type control B6 mice were purchased from Charles River Japan. All mice were housed in the Animal Unit of The Institute of Development, Aging and Cancer (Tohoku University, Sendai, Japan), an environmentally controlled and specific pathogen-free facility. Animal protocols were reviewed and approved by the Tohoku University Animal Studies Committee. All experiments were performed using 12 week-old female mice.

### Surgical procedures (fracturing)

The surgical procedure was performed after inhalation of anesthesia with 2% isoflurane (Forene; Abbott, Wiesbaden, Germany). Closed transverse diaphyseal fractures were created, following intramedullary insertion of 0.3-mm Kirschner wires into the right tibia using the fracture device developed by Bonnarens and Einhorn [21]. The fracture device functioned via a blunt guillotine driven by a 230-g weight dropping from a height of 17 cm. No antibiotic drugs for infection prevention were administered to mice before or after surgery. All mice were allowed to move freely in their cages after surgery.

Animals were euthanized in a carbon dioxide gas chamber 3–28 days after fracture, followed immediately by cardiac perfusion with 4% buffered paraformaldehyde (PFA). The tibia was dissected free of the femur, foot, and overlying skin.

### Radiographic analysis

Lateral radiographs of the right tibiae from B6 and *DAP12* ^*–/–*^ mice were taken just after sacrifice using a soft X-ray apparatus (SRO-M50, Sofron, Tokyo, Japan) and X-ray films (Biomax XAR film, Carestream Health Co., New York, USA) at days 7, 10, 14, 21 and 28 after fracture. Mineralized callus formation and bony bridging at the fracture site were evaluated.

### Micro-computed tomography

Micro-CT imaging was performed at day 14 after fracture (n = 6 for each group). The harvested tibiae were stored in 70% ethanol at 4°C and analyzed using a micro-CT scanner (Scan Xmate-L090; Comscan Techno Co., Ltd, Kanagawa, Japan) operating at a peak voltage of 75 kV and 100 μA. The scanned region included 253 images proximal and distal to the fracture line at a resolution of 10.4 μm per voxel and 516 × 506 pixel image matrices. At the axial slice with the maximal cross-sectional area of the callus, total volume (TV, mm^2^), bone volume fraction (BV/TV, %), trabecular thickness (Tb.Th, μm), trabecular number (Tb.N, 1/mm), and trabecular spacing (Tb.Sp, μm) were evaluated using TRI/3D-BON software (Ratoc System Engineering Co., Tokyo, Japan). The cortical bone area was separated from bone marrow using a fixed threshold of 31 (range, 0–255). The percentage of new cortical shell area (new cortical shell area/total volume, %) was also calculated, as described previously [22], to evaluate woven bone remodeling to lamellar bone.

### Histological analysis

Tibiae were immersed in 4% PFA for 48 h and transferred to 30% sucrose in 0.1 M PBS for 24 h. The tissue was placed in OCT compound (Tissue-Tek; Sakura Finetek, CA, USA), frozen in liquid nitrogen, and stored at -80°C until sectioning. Sagittal cryosections (7 μm) of undecalcified fracture callus were obtained using a cryostat (Bright, Huntingdon, UK) with a disposable microtome blade (S35 Fine, Feather Safely Razor, Osaka, Japan) and tape transfer process (Cryofilm Type II C (10), Section-lab, Hiroshima, Japan). Tissue sections that remained adherent to the tape were placed sample side up on a glass slide and stored at 4°C until use. The sections were stained individually with hematoxylin, alcian blue and tartrate-resistant acid phosphatase (TRAP) using the leukocyte acid phosphatase kit (Sigma-Aldrich, St Louis, MO, USA). Amounts of hematoma, cartilage and newly formed bone were measured using image analysis software (Image J version 1.47t), according to the ASBMR guidelines (n = 6 for each group) [23]. TRAP-positive cells residing on the bone surface were defined and counted as osteoclasts (n = 6 for each group). Osteoclasts with more than three nuclei were defined as mature osteoclasts. The number of osteoclasts was counted under 200-fold magnification in three randomly extracted areas within the periosteal fracture callus, and the average of each area was then calculated.

### Immunohistochemistry

Immunohistochemical localization of the macrophage-specific antigen F4/80 in the callus at days 3 was examined. Undecalcified sections on tape were washed in PBS and placed in 0.5% Triton x100 (Sigma, St. Louis, MO, USA) in PBS for 60 min at 4°C. After pretreatment, the sections were incubated with 10% normal goat serum in PBS for 2 h at room temperature for blocking, followed by overnight incubation in rat polyclonal anti-mouse F4/80 antibody (AbD Serotec Ltd., Oxford, UK) diluted 1:500 at 4°C. Sections were then washed with PBS and incubated with 1:500 goat polyclonal anti-rat secondary antibody conjugated with Alexa Fluor 488 (Abcam, Cambridge, UK) for 60 min at room temperature. Slides were counterstained with hematoxylin and mounted using 50% glycerol in PBS. The number of F4/80-positive cells was counted under 200-fold magnification in three randomly extracted areas within the hematoma close to the fracture area, and the average of each area was then calculated.

### Microscopic imaging

Sections were examined and photographed using an Olympus BX51 microscope and DP73 digital camera (Olympus, Tokyo, Japan) using CellSens Standard software version 1.7 (Olympus, Tokyo, Japan). Alexa Fluor 488-immunostained sections were detected using a U-MNIBA3 filter (Olympus, Tokyo, Japan). CellSens software created an image stack for fluorescence and hematoxylin images.

### Quantitative RT-PCR

Six callus specimens were harvested at days 3, 5, 7, 10, and 14. Muscles and original bones were excluded from the callus. Total RNA was extracted using Trizol (Invitrogen Corp., Carlsbad, California, USA) and an RNeasy Mini Kit (Qiagen, Hilden, Germany). cDNA was synthesized from total RNA using RT buffer, RT random primers, dNTP mix, and Multiscribe reverse transcriptase (Applied Biosystems, Foster city, CA, USA). A total of 9 μl cDNA diluted 1:9 was added to 10 μl Taqman Universal Master Mix II with Uracil-N glycosylase (Applied Biosystems, Foster City, CA, USA). Real-time amplification of the genes was performed using 1 μl ready-to-use Taqman Gene Expression Assays (Applied Biosystems) for *Il1b*, *Il6*, *Tnf*, *Col1a1*, *Col2a1*, *Col10a1*, and *Actb* as an endogenous control (assay IDs: Mm00434228_m1, Mm00446190_m1, Mm00443260_g1, Mm00801666_g1, Mm01309565_m1, Mm00487041_m1, and Mm00607939_s1). Relative gene expression data were analyzed using the delta-delta-Ct method with PCR-efficiency correction using StepOne software version 2.2.2 (Applied Biosystems) [24].

### Statistical analysis

Statistical analyses were performed using PASW Statistics 18.0 (SPSS, Chicago, IL, USA). All data were expressed as the means ± SE. The statistical significance of the differences between values was evaluated using the Student’s t-test. Differences were considered significant at p<0.05.

## Results

### Radiographic findings

**Fig 1:**
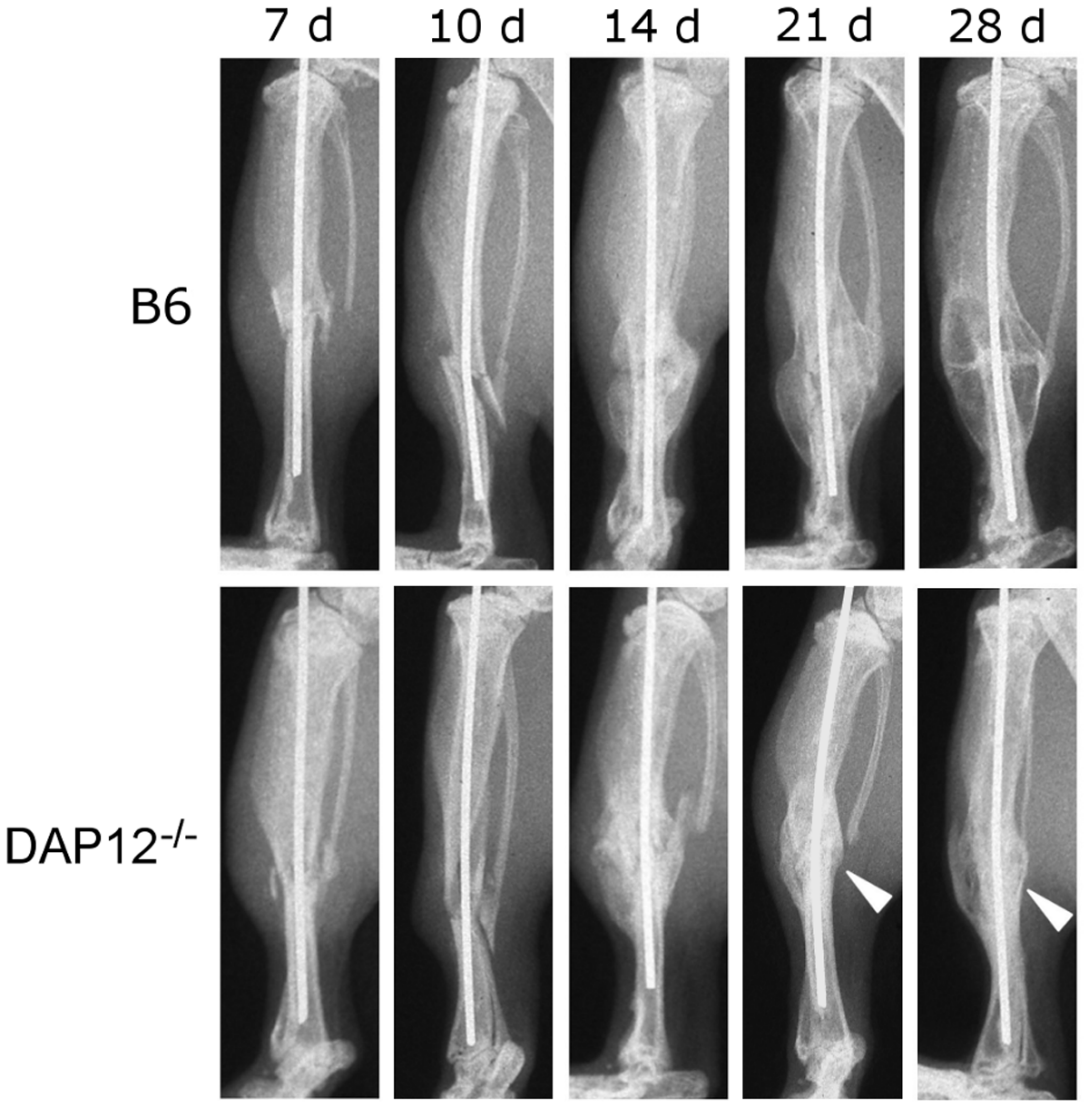
Radiographic images of fractured tibiae in B6 and *DAP12* ^*–/–*^ mice. *DAP12* ^*–/–*^ mice showed lower callus volume and radiolucency at days 21 and 28 compared with B6 mice (arrow head).

Representative X-ray images of the fracture models are shown in Fig 1. In B6 and *DAP12* ^*–/–*^ mice, mineralized callus formation was first detected at day 14. Bony bridging of the fracture site was complete by day 21 in both groups. Compared with B6 mice, *DAP12* ^*–/–*^ mice showed lower callus volume and radiolucency at days 21 and 28. These findings suggested that Original cortical bone resorption was delayed in *DAP12* ^*–/–*^ mice at day 28. Non-union was not observed.

### Micro-CT findings and micro-architectural analysis

**Fig 2:**
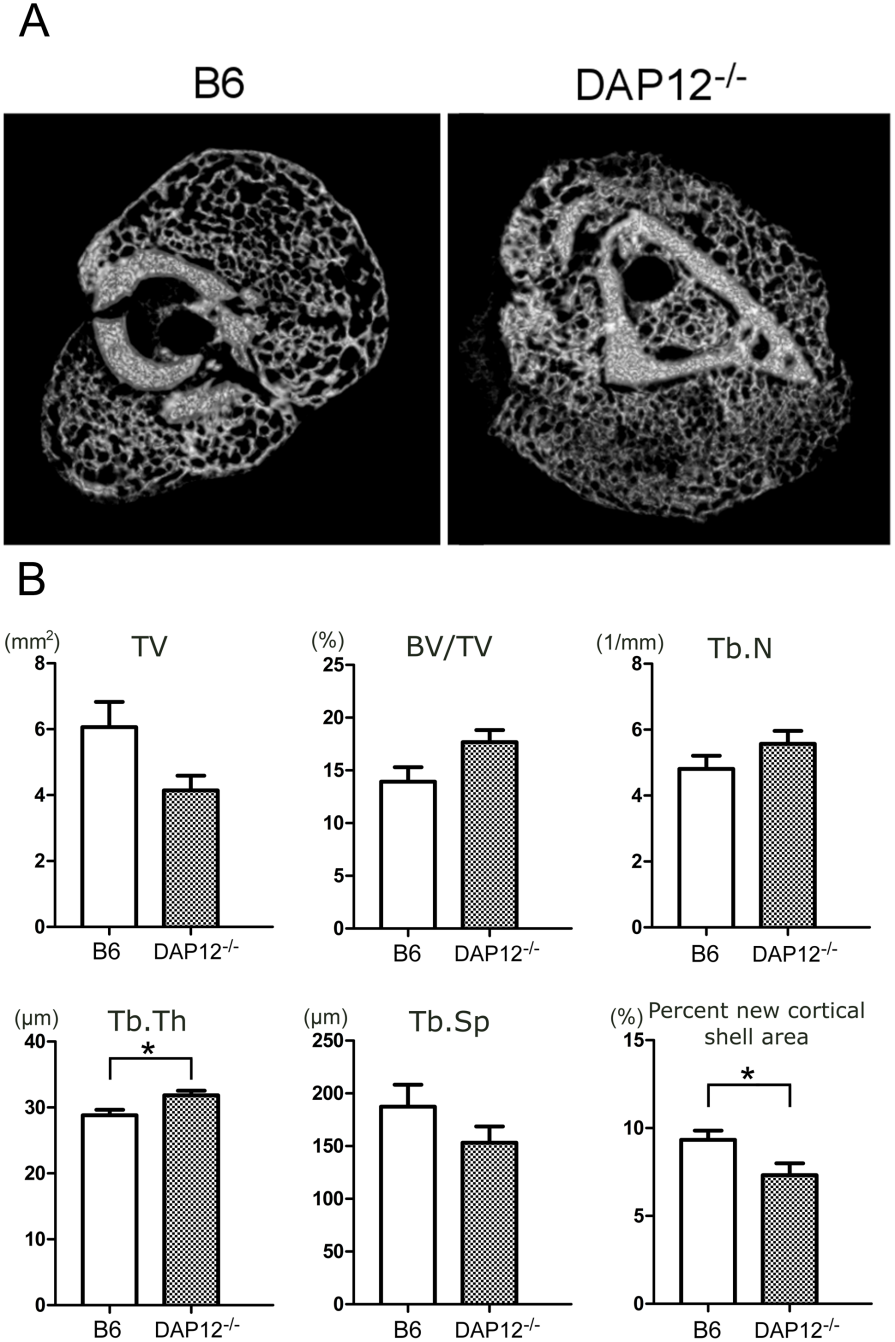
Micro-CT image analysis of fracture callus at day 14 in B6 and *DAP12* ^*–/–*^ mice. (A) Representative axial images of fracture callus in B6 and *DAP12* ^*–/–*^ mice. Dense trabecular structure and deteriorated cortical shell formation on the surface in *DAP12* ^*–/–*^ mice compared with B6 mice. (B) Structural parameter analysis of fracture callus of B6 and *DAP12* ^*–/–*^ mice. Trabecular thickness was significantly larger in *DAP12* ^*–/–*^ mice than in B6 mice, and the percentage of new cortical shell area was significantly lower in *DAP12* ^*–/–*^ mice. There were no significant differences in TV, BV/TV, Tb.N or Tb.Sp. *p<0.05.

In the reconstructed micro-CT images at day 14, *DAP12* ^*–/–*^ mice showed dense trabecular structures and deteriorated cortical shell formation on the callus surface (Fig 2A), while B6 mice did not.

Quantitative structural analysis of fracture callus is shown in Fig 2B. Trabecular thickness was significantly greater in *DAP12* ^*–/–*^ mice than in B6 mice (32 ± 0.71 μm vs. 29 ± 0.83 μm, respectively; p < 0.05).

The percentage of new cortical shell area was significantly lower in *DAP12* ^*–/–*^ mice than in B6 mice (7.3 ± 0.67% vs. 9.3 ± 0.53%, respectively; p < 0.05), which was indicative of suppressed formation of new cortical shell in *DAP12* ^*–/–*^ mice (Fig 2B).

### Histological analysis

**Fig 3:**
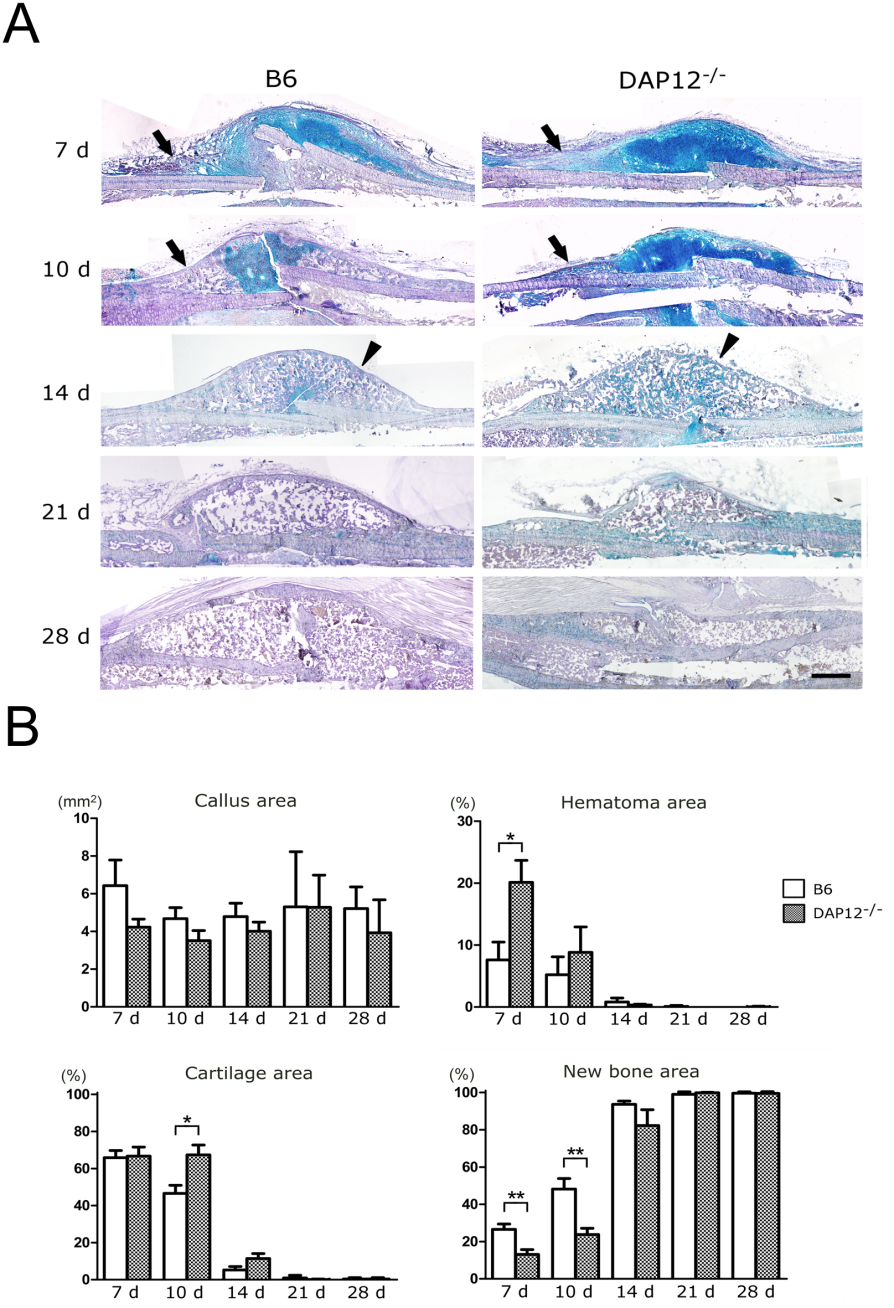
Histological images of fracture healing. (A) Alcian blue-stained sections. Scale bar = 500 μm. *DAP12* ^*–/–*^ mice showed more residual cartilaginous tissue and delayed woven bone formation (arrow) at day 10. Substantially impaired cortical shell formation on the callus surface was observed in *DAP12* ^*–/–*^ mice at day 14 (arrowhead). *DAP12* ^*–/–*^ mice showed residual cartilage matrix in the peripheral callus, less original cortex absorption and delayed bone remodeling at day 28. (B) Quantitative histomorphometric analysis of the fractured callus in B6 and *DAP12* ^*–/–*^ mice. There was no significant difference in callus volume between *DAP12* ^*–/–*^ and B6 mice. Hematoma area was significantly larger in *DAP12* ^*–/–*^ mice than in B6 mice at day 7. The newly formed bone area was significantly smaller in *DAP12* ^*–/–*^ mice than in B6 mice at days 7 and 10. Cartilage area was significantly larger in *DAP12* ^*–/–*^ mice than in B6 mice at day 10. *p<0.05, **p<0.01.

Alcian-blue staining showed cartilaginous tissue in the fractured callus, indicating that the process of cartilaginous absorption and woven bone formation proceeded from the peripheral to the central callus in both B6 and *DAP12* ^*–/–*^ mice at day 10. Compared with B6 mice, *DAP12* ^*–/–*^ mice showed more residual cartilaginous tissue and delayed woven bone formation (Fig 3A). In *DAP12* ^*–/–*^ mice, alcian-blue staining was still observed in the peripheral callus at day 10. Fourteen days after fracture, cortical shell formation on the callus surface was observed in B6 mice. In contrast, shell formation was much less prominent in *DAP12* ^*–/–*^ mice (Fig 3A). Fracture gaps were connected by bony callus, and absorption of the original cortex started in B6 mice at day 21. On the other hand, *DAP12* ^*–/–*^ mice showed residual cartilage matrix in the peripheral callus, less original cortex absorption and delayed bone remodeling (Fig 3A).

Quantitative histomorphometric analysis was performed (Fig 3B). No significant difference in the amount of fractured callus was observed among B6 mice and *DAP12* ^*–/–*^ mice. At day 7, *DAP12* ^*–/–*^ mice exhibited a significantly large hematoma area (20.1 ± 3.5% vs. 7.6 ± 2.9%, respectively; p < 0.05) and a small, newly formed bone area (13.1 ± 2.6% vs. 26.5 ± 2.9%, respectively; p < 0.01). Ten days after fracture, *DAP12* ^*–/–*^ mice exhibited a significantly large amount of cartilage area (67.4 ± 5.3% vs. 46.6 ± 4.4%, respectively; p < 0.05) and a small, newly formed bone area (23.8 ± 3.4% vs. 48.2 ± 5.6%, respectively; p < 0.01). Fourteen days after fracture, most of the callus area was replaced by a newly formed bone area, although there was no significant difference among *DAP12* ^*–/—*^ and B6 mice (82.3 ± 8.5% vs. 93.6 ± 1.8%, respectively; n.s.).

**Fig 4:**
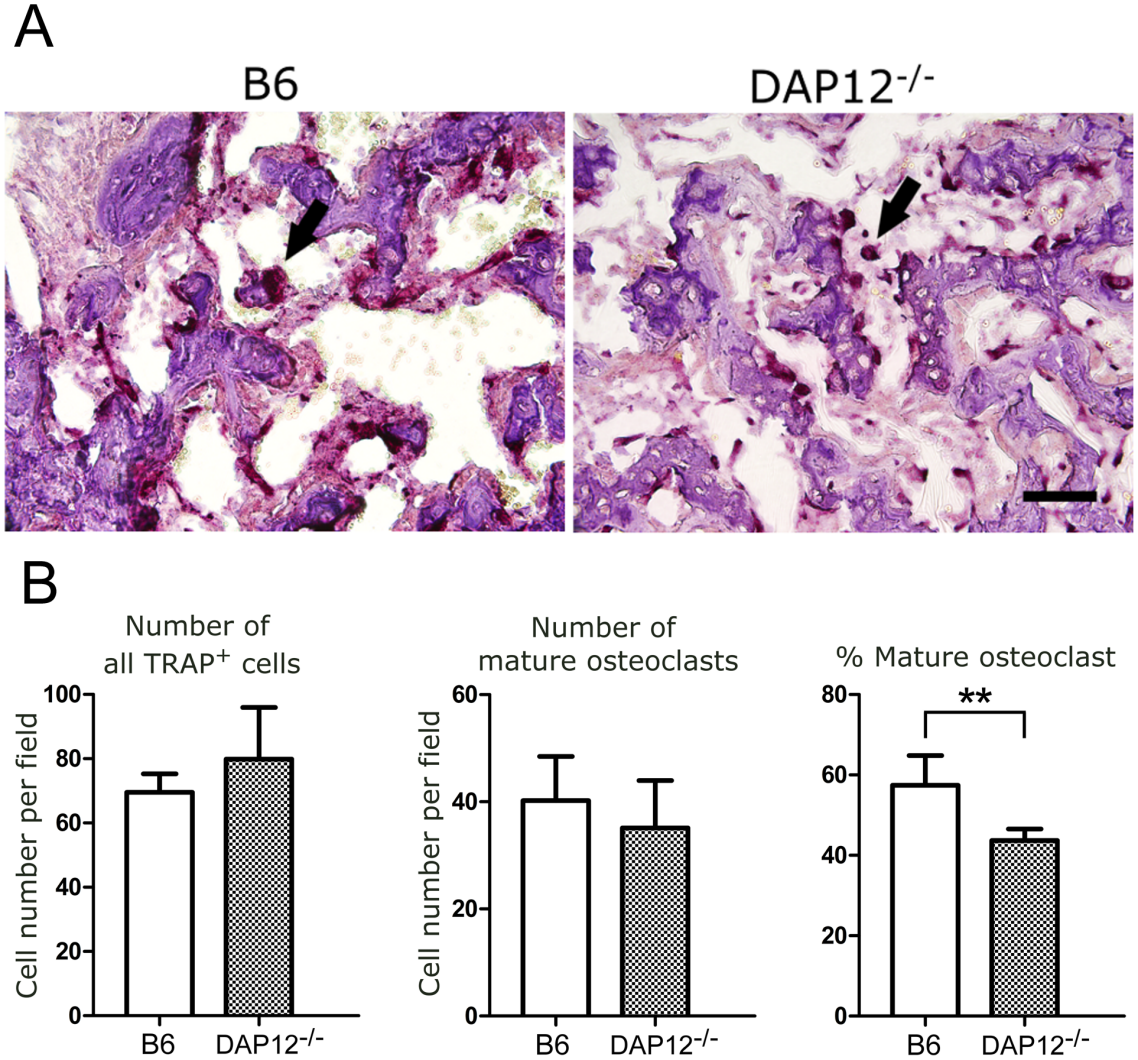
Histochemical analysis for TRAP staining. Histochemical analysis for TRAP indicates osteoclasts (arrow) in the fracture callus at day 14. Scale bar = 50 μm. (A) *DAP12* ^*–/–*^ mice contained smaller and less matured osteoclasts than did B6 mice. (B) Quantitative analysis of TRAP-positive cells. The percentage of mature osteoclasts (more than three nuclei) was significantly smaller in *DAP12* ^*–/–*^ mice than in B6 mice. **p<0.01.

TRAP staining showed osteoclasts that appeared in newly formed bone at day 14 in both groups; however, those observed in *DAP12* ^*–/–*^ mice were smaller and less mature compared with B6 mice (Fig 4A). Quantitative histomorphometric analysis was performed (Fig 4B). There was no significant difference in the number of TRAP-positive cells or mature osteoclasts between B6 and *DAP12* ^*–/–*^ mice. However, the ratio of mature osteoclasts in TRAP-positive cells was significantly decreased in *DAP12* ^*–/–*^ mice compared with B6 mice (43.7 ± 1.3% vs. 57.4 ± 3.3%, respectively; p < 0.01) (Fig 4B).

### Immunohistochemistry

**Fig 5:**
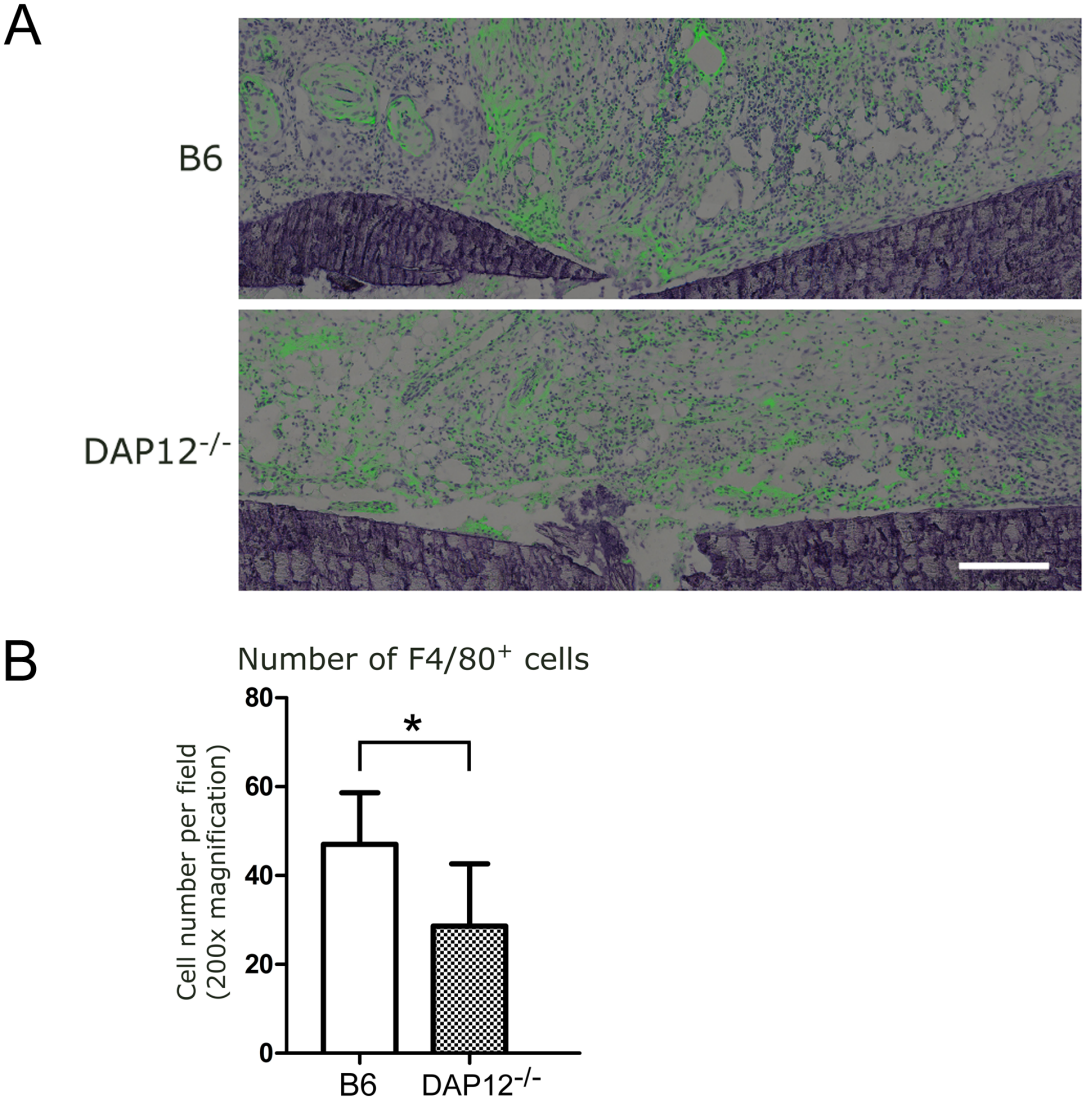
Immunostaining using an anti-F4/80 antibody. (A) Representative images of fracture hematoma at day 3. Scale bar = 200 μm. *DAP12* ^*–/–*^ mice showed less fluorescence from F4/80-positive cells in fracture hematoma. (B) Quantitative analysis of F4/80-positive cells. The number of F4/80-positive cells was significantly decreased in *DAP12* ^*–/–*^ mice than in B6 mice. *p<0.05.

Lower F4/80-positive cellular invasion into the fractured hematoma was observed in *DAP12* ^*–/–*^ mice compared with B6 mice at day 3 (Fig 5A). Quantitative histomorphometric analysis was performed. The number of F4/80-positive cells was significantly decreased in *DAP12* ^*–/–*^ mice compared with B6 mice (28.6 ± 5.7% vs. 47.1 ± 5.2%, respectively; p < 0.05; Fig 5B).

### RT-PCR

**Fig 6:**
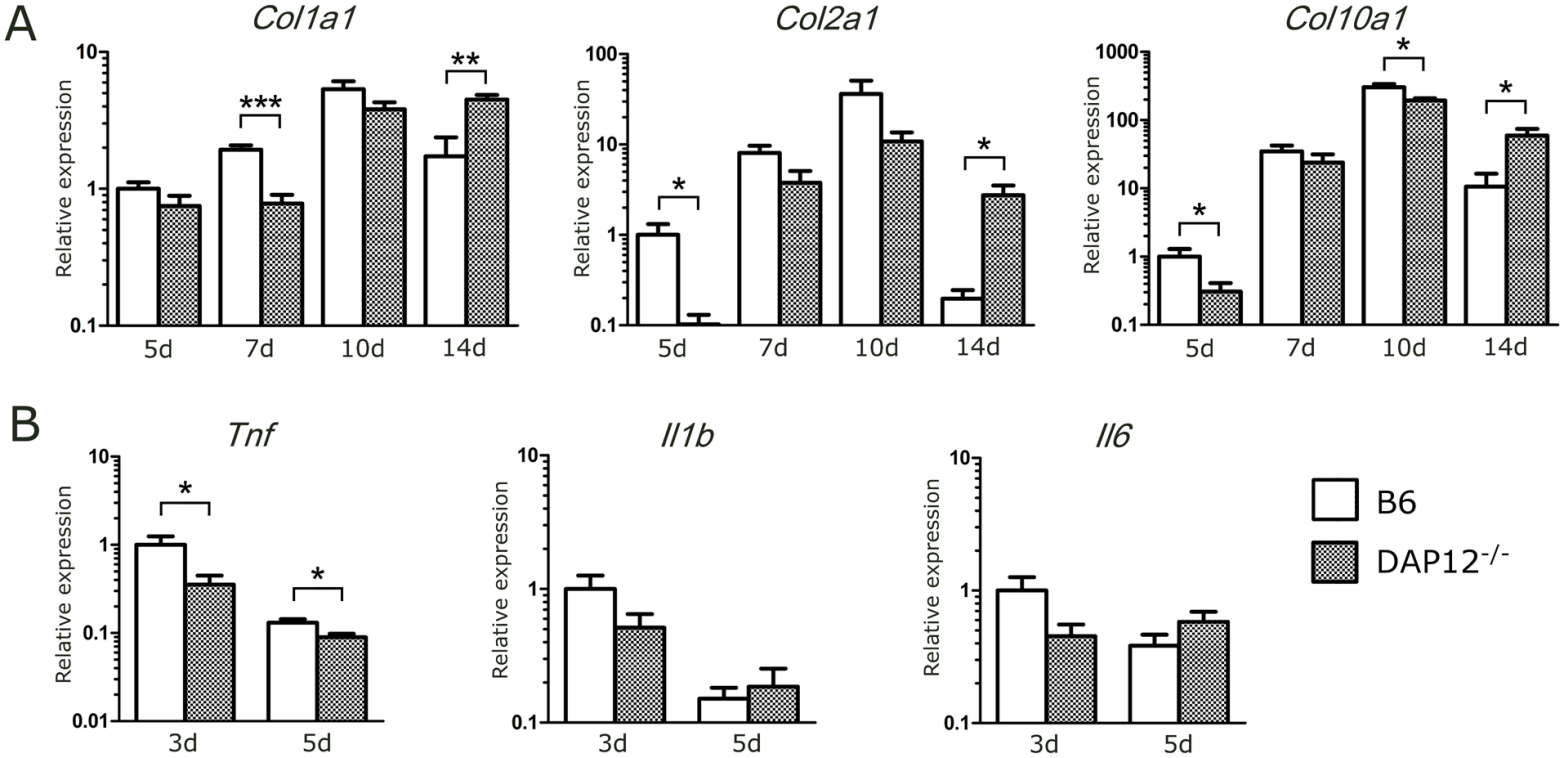
mRNA expression of osteogenic, chondrogenic and proinflammatory genes. (A) Expression levels of *Col1a1*, *Col2a1*, and *Col10a1* in the fracture callus. *DAP12* ^*–/–*^ mice demonstrated a delayed increase in *Col1a1* gene expression. *DAP12* ^*–/–*^ mice showed significantly lower expression of *Col1a1* at day 7 and significantly higher expression at day 14. In *DAP12* ^*–/–*^ mice, the expression of *Col2a1* and *Col10a1* was significantly lower at day 5 and significantly higher at day 14. *DAP12* ^*–/–*^ mice showed delayed decreases in *Col2a1* and *Col10a1* gene expression. (B) Expression levels of *Tnf*, *Il1b*, and *Il6* in fracture hematomas. The expression of *Tnf* was significantly lower in *DAP12* ^*–/–*^ mice at days 3 and 5. There were no significant differences in the expression of *Il1b* or *Il6*. *p<0.05, **p<0.01, ***p<0.001.

The expression of the osteogenic gene *Col1a1* increased progressively until day 10 post-fracture, and it was significantly lower at day 7 in *DAP12* ^*–/–*^ mice compared with B6 mice. Although the expression of *Col1a1* decreased at day 14 in B6 mice, it continued to increase in *DAP12* ^*–/–*^ mice. Expression of the chondrogenic genes *Col2a1* and *Col10a1* increased in both B6 and *DAP12* ^*–/–*^ mice until 10 days post-fracture. At day 5, expression of *Col2a1* and *Col10a1* was significantly higher in B6 mice. At day 10, expression of *Col10a1* was significantly higher in B6 mice. At day 14, expression of these genes in B6 mice decreased significantly, becoming significantly lower than that in *DAP12* ^*–/–*^ mice (Fig 6A).

There was a decrease in *Tnf* gene expression at days 3 and 5 in *DAP12* ^*–/–*^ mice; on the other hand, there were no significant differences in *Il1b* or *Il6* gene expression between the two groups (Fig 6B).

These data suggested that the early processes of fracture repair, such as the initial inflammatory response in hematoma, cartilaginous callus formation and resorption, and subsequent woven bone formation, were delayed in *DAP12* ^*–/–*^ mice compared with B6 mice.

## Discussion

The present study explored the effect of the defect in DAP12 on fracture healing in mice. Our results demonstrated that the process of fracture healing in *DAP12* ^*–/–*^ mice showed a decreased inflammatory response during the early phase, inhibition of cartilaginous callus formation and resorption during the middle phase, and inhibition of woven bone remodeling during the late phase.

In the early phase of fracture repair, primary bleeding within fracture sites develops into a hematoma, and inflammatory cells that infiltrate the hematoma (including macrophages) induce inflammatory cascades through the secretion of various cytokines [25]. Macrophages produce pro-inflammatory cytokines such as interleukin(IL)-1, IL-6, and tumor necrosis factor (TNF)-α [26]. These factors recruit additional inflammatory cells and initiate repair cascades [27,28]. In this study, *DAP12* ^*–/–*^ mice showed inhibited inflammation in the early phase of fracture repair, such as lower gene expression of *Tnf* and lower cellular density of monocytes and macrophages in the fracture hematoma. A previous study reported that DAP12 signaling plays an important role in macrophage differentiation and activation [29], and its deficiency presumably inhibits macrophage migration into hematomas and cytokine secretion by these cells, leading to cartilaginous callus and woven bone formation.

During the middle phase of fracture repair, in histological analysis, *DAP12* ^*–/–*^ mice showed delayed cartilaginous callus resorption and woven bone formation. RT-PCR analysis showed that *Col1a1* expression was significantly higher at day 7 in B6 mice compared with *DAP12* ^*–/–*^. At day 14 *Col1a1* expression was significantly higher in *DAP12* ^*–/–*^ mice. Expression of *Col2a1* and *Col10a1* was significantly higher in B6 mice at day 5. At day 14, expression of *Col2a1* and *Col10a1* was significantly higher in B6 mice. These findings of gene expression also indicated delayed woven bone formation and cartilaginous callus resorption in *DAP12* ^*–/–*^ mice. It was previously believed that cartilaginous callus resorption was performed by chondroclasts, which exhibit similar morphologies to osteoclasts and express classic osteoclastic markers [30]. However, in contrast to our results, recent studies examining fracture repair in osteoclast-deficient or osteoclast-inhibited animals using incisors [14], anti-RANK ligand antibody [31], recombinant osteoprotegerin [17], or zoledronic acid [15] showed no delay in cartilaginous callus resorption or woven bone formation. These results suggest that osteoclasts may be redundant in the remodeling of soft callus. Instead, matrix metalloproteinases (MMPs) and TNF-α may be responsible for soft callus remodeling. MMP-9 is involved in extracellular matrix degradation, is expressed in inflammatory cells and osteoclasts, and localizes to cells of the chondro-osseus junction [32,33]. TNF-α may regulate MMP-9, MMP-14 and angiogenic factor [34] and induce apoptosis of hypertrophic chondrocytes through upregulation of proapoptotic genes, such as caspase-3, -8, -9, and TRAIL [28,35]. In this study, suppression of inflammatory cells may decrease the secretion of MMP-9 and TNF-α, causing delays in cartilage resorption and subsequent woven bone formation.

During the late phase of fracture repair, cortical shells originate from the external callus and reconstruct original bone structures through remodeling of woven bone to lamellar bone [36]. *DAP12* ^*–/–*^ mice showed impaired cortical shell formation on the callus surface, and callus volume tended to be smaller. Li et al. investigated fracture repair in rats treated with incadronate and found that cortical shell formation was impaired similarly, but callus volume was larger [5], in contrast to our study. They suggested that greater callus formation was not caused by accelerated bone formation but by inhibited bone resorption, because the callus volume in rats treated with incadronate was similar 2 weeks after fracture but increased 4 weeks after fracture compared with the control group [18]. Cao et al. treated ovariectomized rats with alendronate and investigated fracture healing [37]. Ovariectomized rats showed increased cortical shell formation and smaller callus volume; on the other hand, rats treated with alendronate showed decreased cortical shell formation and larger callus volume, and their mechanical strength was comparable to the control group. They suggested that higher bone turnover results in early remodeling and volume reduction of the fracture callus, and that the larger callus volume observed in rats treated with alendronate was an adaptation that compensated for delays in woven bone remodeling into lamellar bone, which is superior structurally and mechanically. In the *DAP12* ^*–/—*^mouse fracture model, inflammatory suppression during the early phase may inhibit callus formation and result in smaller callus volume. *DAP12* ^*–/–*^ mice are osteopetrotic and their intramedullary space putatively tends to be smaller; therefore, stability may be improved using the same intramedullary wire as in B6 mice and could decrease the callus volume. Further studies are required to determine whether smaller callus volumes in *DAP12* ^*–/–*^ mice are due to mechanically higher strength compared with bisphosphonate-treated mice or to inhibited callus formation. Bisphosphonates may stimulate proliferation of osteoblasts and inhibit apoptosis of osteoblasts, in addition to having an inhibitory effect on osteoclasts [38]. In animal fracture models treated with bisphosphonates, increased bone formation by osteoblasts may be responsible for the increased callus formation.

It is important to note the limitations of the present study. First, wild-type B6 mice (control) were not littermates of the *DAP12* ^*–/–*^ mice. This difference in background may have caused a difference in fracture healing and the influence of the microbiome on the inflammatory response required for fracture healing.

In conclusion, our study showed that DAP12 plays a significant role in the initial inflammatory response and subsequent woven bone remodeling into lamellar bone during fracture repair. These results increase our understanding of the fracture healing process and of the physiological role of DAP12.
